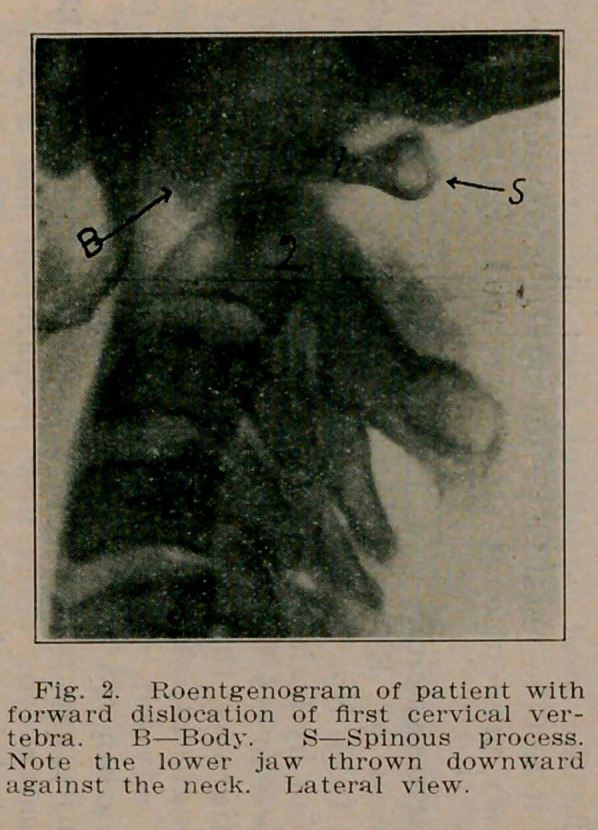# Dislocation of First Cervical Vertebra

**Published:** 1916-02

**Authors:** 


**Dislocation of First Cervical Vertebra.** A. F. Tyler of Omaha in Western Med. Rev. Dec. (cuts by courtesy of author). Three cases have been seen in the last year, without death or even paralysis, but with stiff neck and downward tipping of the chin. The condition is that produced in hanging, but with escape of spinal cord from injury by odontoid process. Two cases were due to being thrown from wagons, the third was ascribed by the patient to osteopathy—the movements consisting in partial rotation of the head with a forward thrust. This dislocation implies either rupture of the transverse ligament, fracture of odontoid, or slipping of the latter from the ligament. A few cases of reduction have been reported, but on account of the danger of instant death, none of these have consented to an attempt at reduction.

## Figures and Tables

**Fig. 1. f1:**
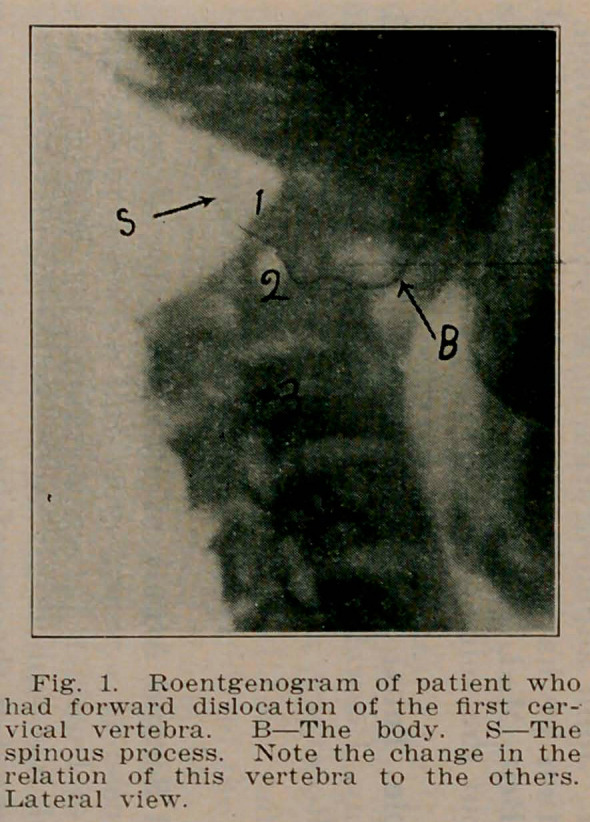


**Fig. 2. f2:**